# Metabolic memory in mitochondrial oxidative damage triggers diabetic retinopathy

**DOI:** 10.1186/s12886-018-0921-0

**Published:** 2018-09-24

**Authors:** Zhaoge Wang, Haixia Zhao, Wenying Guan, Xin Kang, Xue Tai, Ying Shen

**Affiliations:** Center of Myopia, the Affiliated Hospital of Inner Mongolia Medical University, 1 Tongdao North Street, Hohhot, 010050 China

**Keywords:** Diabetic retinopathy, Metabolic memory, ROS, Mitochondria, Apoptosis

## Abstract

**Background:**

Diabetic retinopathy (DR) is a microvascular complication induced by high blood glucose. This study was conducted to investigate the effect of metabolic memory on mitochondrial oxidative damage-induced DR.

**Methods:**

Rat retinal endothelial cells (rRECs) were isolated from SD rats and treated with high glucose (20 mM) for various times and then cultured in normal glucose (5.6 mM) medium for 2 days. The cells were assayed for the expression of respiratory chain complexes *cytochrome c oxidase* subunit *1* (CO1) and NADPH-1 using RT-PCR, mitochondrial membrane potentials and reactive oxygen species (ROS) production using flow cytometry and apoptosis using Annexin V/PI flow cytometry.

**Results:**

rRECs displayed like short spindles after cultured for 9–10 days and reached 100% confluency. Compared with the control grown in normal glucose (5.6 mM) medium, rRECs exposed to high glucose medium for 3, 12 and 24 h had significantly increased mRNA levels of CO1 and NAPDH-1 even after being shifted back to normal glucose medium. They also had lower mitochondrial membrane potential (89.13% vs 78.21%, *p* < 0.05), cytochrome C level (1 in control vs 0.25 after 24 h exposure to high glucose, *p* < 0.05 and higher ROS production (2.77% in control vs 9.00% after 12 h exposure to high glucose, *p* < 0.05) and apoptosis (7.15% in control vs and 29.91% after 24 h exposure to high glucose, *p* < 0.05).

**Conclusion:**

It is likely that mitochondrial oxidative damage triggers metabolic memory via ROS overproduction, leading to diabetic retinopathy.

## Background

Diabetic retinopathy (DR) is a microvascular complication induced by high blood glucose. It is the main cause of blindness in the working population aged 20 to 65 years old [1, 2]. Due to its the high incidence and severe complications, DR has become a priority for blindness prevention and treatment in [3, 4]. Intensive studies have been conducted to investigate DR in diabetic complications [5]. As a consequence, a special phenomenon hyperglycemic memory or metabolic memory has been discovered, which occurs when human cells have prolonged exposure to hyperglycemia conditions even after hyperglycemic control is therapeutically achieved [6, 7]. As a result, disease may continue to occur or progress after the patient’s blood glucose has been controlled for a long period of time and cells may continue to be damaged after the high glucose environment has been removed [8, 9]. For diabetic patients, metabolic memory probably is an important cause of continuing disease progress after their blood glucose is controlled.

When the balance of oxidation-antioxidation system is broken, excessive reactive oxygen species (ROS) is produced, resulting in cytotoxicity and oxidative stress. The excessive ROS is mainly produced in the mitochondrial respiratory chain [10]. Since mitochondrial DNA (mtDNA) is very close to where ROS is produced, and there is no effective DNA repair system in mtDNA as in nuclear DNA, mtDNA is very vulnerable to ROS attack. Once damaged, the expression of mitochondrial genes would be compromised, leading to reduced mitochondrial membrane potential and increased apoptosis, which in turn increases ROS production, and subsequently continued ROS overproduction [11]. Previous study showed that there was mtDNA oxidative damage in the retinal vessels and ROS was excessively produced in the early stage of DR [12]. Since metabolic memory is a refractory phenomenon in the progress of DR, we speculated that this vicious cycle of ROS production continuously promotes the process of metabolic memory, leading to the mtDNA oxidative damage in retinal blood vessels. In recent years, studies have shown that oxidative stress is responsible for complications of diabetes, including DR and is closely related to metabolic memory [6, 13]. Therefore, oxidative stress is likely involved in DR metabolic memory.

To better understand the effect of metabolic memory on DR with respect to mitochondrial oxidative damage, we investigated the cellular damage and functions using rat retinal endothelial cells (rRECs) by exposing the cells to high glucose to simulate metabolic memory. The work would provide insight into how mitochondrial oxidative damage triggers metabolic memory and promotes the development of DR via the excessive ROS production.

## Methods

### Isolation of rRECs

SD rats (purchased from Yingniurui Biotech, Wuxi, China) were sacrificed by cervical dislocation and the eyeballs were isolated. The retinas were collected, washed with the D-Hank’s solution and cut into pieces of 1 × 1 mm in size. The tissues were incubated in 0.25% trypsin solution (Keygentec, China) at 37 °C for 30 min and filtered through a nylon sieve with 30 μm pore size. The released cells were pelleted by centrifugation at 1000 rpm for 5 min, and inoculated into culture flasks containing Dulbecco’s Modified Eagle’s Medium (DMEM) (HZSJQ Biotech, Hangzhou, China) with 20% fetal bovine serum (FBS, *Bi*oligo, Shanghai, China) and cultured at 37 °C. 24 h later, the medium was refreshed and non-adherent cells were removed. One day after culture, radial cells were grown out of the vessel fragments and 3 days later the cells become visible. The cells were then passaged every 3 days and were used for experiments at the third passage. All animal experimental protocols were approved by Inner Mongolia Medical University. All animals received humane care in compliance with the ‘Principles of Laboratory Animal Care’ formulated by the National Society for Medical Research and the ‘Guide for the Care and Use of Laboratory Animals’ prepared by the Institute of Laboratory Animal Resources and published by the National Institutes of Health (NIH Publication No. 86–23, revised 1996).

### Treatment of rRECs

The cells at 100% confluency were digested with 2% trypsin and suspended in DMEM containing with 20% FBS and 5.6 mM glucose. The cells were then pelleted by centrifugation at 1000 rpm for 3 min and inoculated into the DMEM medium containing normal level of glucose (5.6 mM) or high level of glucose (20 mM) for different times. The cells grown in the high-glucose medium were then transferred to normal-glucose medium to grow for another 2 days before being used for assays. The concentration and duration of glucose treatments were selected based on an early study [14, 15], where up to 30 mM glucose was used to create a hyperglycemic condition in endothelial cells.

### RT-PCR

Total RNA was extracted using Trizol reagents (Invitrogen, USA) according to the manufacturer’s instructions and reversely transcribed into cDNA in a total volume of 10 μl using the High Capacity cDNA Transcriptase Reverse kit (Applied Biosystems by Life Technologies, Carlsbad, California, USA) according to manufacturer’s recommendations. The resulting cDNA amplified using 2 x GoldStar Taq MasterMix (CWBiotech, Beijing, China) in a total volume of 20 μl. Amplification cycling conditions were 3 min at 95 °C followed by 30 cycles, each one consisting of 10 s at 95 °C and 30 s at 50.6 °C, with a final extension of 30 S at 72 °C. RT-qPCR was performed on the 7900HT Fast Real-Time PCR system using TaqMan gene expression assays probes (Applied Biosystems). The primers used for *cytochrome c oxidase* subunit *1 (*CO1) were F: GTAACTACCTACTGCCTCTG, R: CACCACCATACATCCTAA), NADPH-1 F: TGTCCAGGGTGGGTAAGA, R: TGGGAGGAATCGTGAAGT. Human glyceraldehyde-3-phosphate dehydrogenase (GADPH) was used as an internal control (primers: F: GCAAGTTCAACGGCACAG, R: CGCCAGTAGACTCCACGAC). Samples were run in triplicate and the mean value was calculated for each case.

The data were managed using the Applied Biosystems software RQ Manager v1.2.1. Relative expression was calculated by using comparative Ct method and obtaining the fold change value (2^−ΔΔCt^) according to previously described protocol [16].

### Western blot analysis

After different treatments, the cells were harvested, washed twice with cold PBS and lysed with RIPA buffer that containing protease and phosphotase inhibitors cocktail (Roche, UK). The supernatants were collected after centrifugation at 12000 rpm for 20 min. The protein was applied to polyacrylamide gel electrophoresis (SDS-PAGE), transferred to a PVDF membrane, and then detected by goat anti-rat cytochrome C antibody (Abcam, USA) and goat anti-mouse horseradish peroxidase (HRP)-conjugated secondary antibodies (CWBoitech, Beijing, China) before visualization with ChemiDocXRS+ (Biorad, USA). The intensity of blot signals was quantitated using ImageQuant TL analysis software (General Electric, UK).

### Analysis of mitochondrial membrane potential

Cells were harvested, washed twice with cold PBS and stained with diluted JC-1 solution (Molecular Probe by life Technology, USA) according to the manufacturer’s instructions. After incubation at 37 °C in 5% CO_2_, the cells were washed twice with incubation buffer and loaded to a cytometer (Bection Dikinson, USA) for analysis of mitochondrial membrane potential.

### Analysis of mitochondrial ROS

Cells were harvested, washed twice with cold PBS and reacted to dichloro-dihydro-flurescein diacetate (DCFH-DA, Molecular Probes, USA) to detect mitochondria-specific ROS using MitoFLuor Red589 (MFL, Molecular Probes) according to manufacturer’s instructions. The cells were analyzed on a cytometer (Bection Dikinson, USA) and the florescence was detected at an emission wavelength of 525 nm and excitation wavelength of 488 nm.

### Detection of apoptosis by flow cytometry

Cells were collected and suspended in PBS, labeled with Annexin V and propidiumiodide (PI) following the manufacturer’s instructions (Biosea Biotechnology, Beijing, China). Flow cytometry (Bection Dikinson, USA) was used to assess the apoptotic cells. The quantitation of apoptotic cells was calculated by CellQuest software.

### Statistical analysis

All data were expressed as means ± standard derivation (s.d.) obtained from at least three independent experiments. Means were compared using the student’s t-test or one-way ANOVA with the corresponding post-test. A *p*-value ≤0.05 was considered statistically significant. Statistical analyses were performed using GraphPad Prism 5.0 (GraphPad Software Inc., USA).

## Results

### Culture of rRECs

**Fig. 1:**
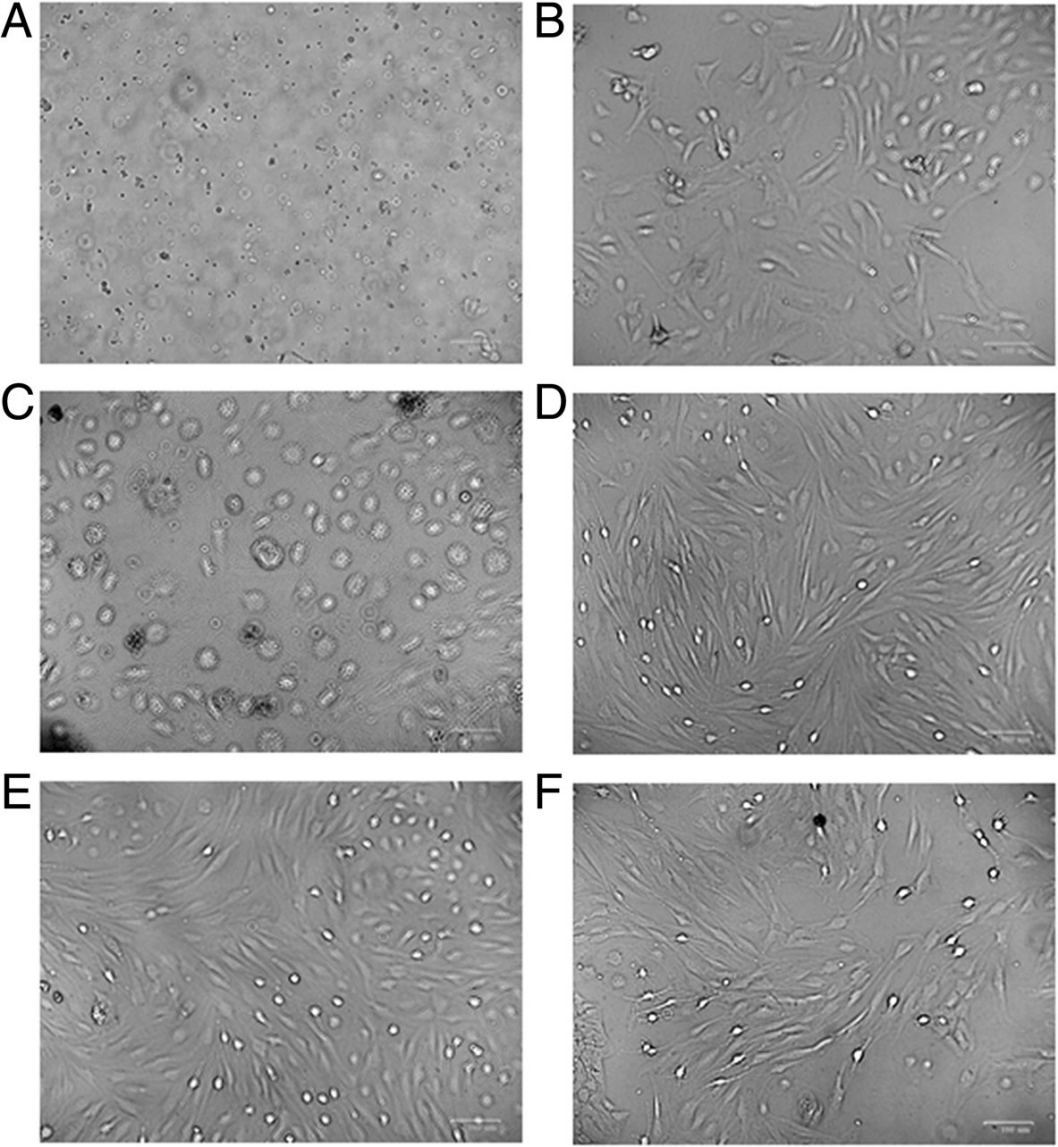
Cultured rat retinal endothelial cells. **a**-**d**, primary cells on day 1, 3, 7 and 9; **e**-**f**, the first passage cells on day 1 and 5

To obtain rREC culture, the retinal tissue was digested with collagenase and cells were isolated through filtration. One day after culture, radial cells were grown out of the vessel fragments and 3 days later the cells become visible. Seven days later the cells were long spindle-shaped and 9 days later they become short spindle-shaped. After passage, the cells were fully expended and grew faster as long spindle (Fig. 1). These cells were used for subsequent experiments.

### High glucose down-regulated the transcription of CO1 and NADPH-1

**Fig. 2:**
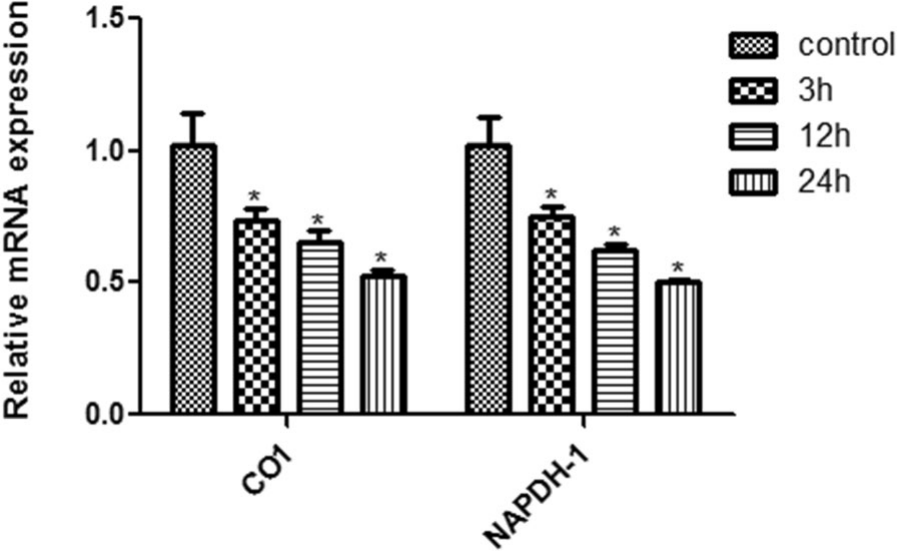
Relative mRNA levels of mitochondrial complex CO1 and NAPDH-1 in rRECs after exposure to high glucose (20 mM) for different time. * denotes significant difference vs control. The bars represent std errors

We then examined the mRNA levels of CO1 and NADPH-1 in the rRECs. The results showed that compared with control cells that were grown at normal level of glucose, the expression of CO1 and NADPH-1 was gradually and significantly reduced after the cells were exposed to high-glucose from 3 to 24 h (Fig. 2). After 24 h exposure, the mRNA levels of the two genes were about half of control (Fig. 2).

### High glucose down-regulated the level of cytochrome C

**Fig. 3:**
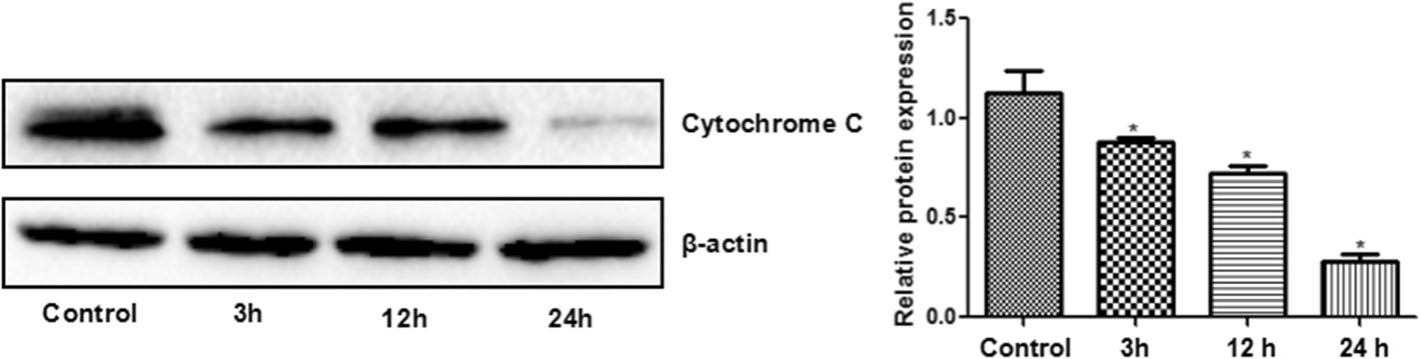
Protein expression of cytochrome C in rRECs after exposure to high glucose (20 mM) for different time. Right panel: representative Western blots, left panel: relative expression levels. * denotes significant difference vs control. The bars represent std errors

Similarly, compared with the control, 3 h, 12 h, and 24 h exposure to high glucose significantly down-regulated the protein expression of cytochrome C in the rRECs (Fig. 3).

### High glucose reduced mitochondrial membrane potential

**Fig. 4:**
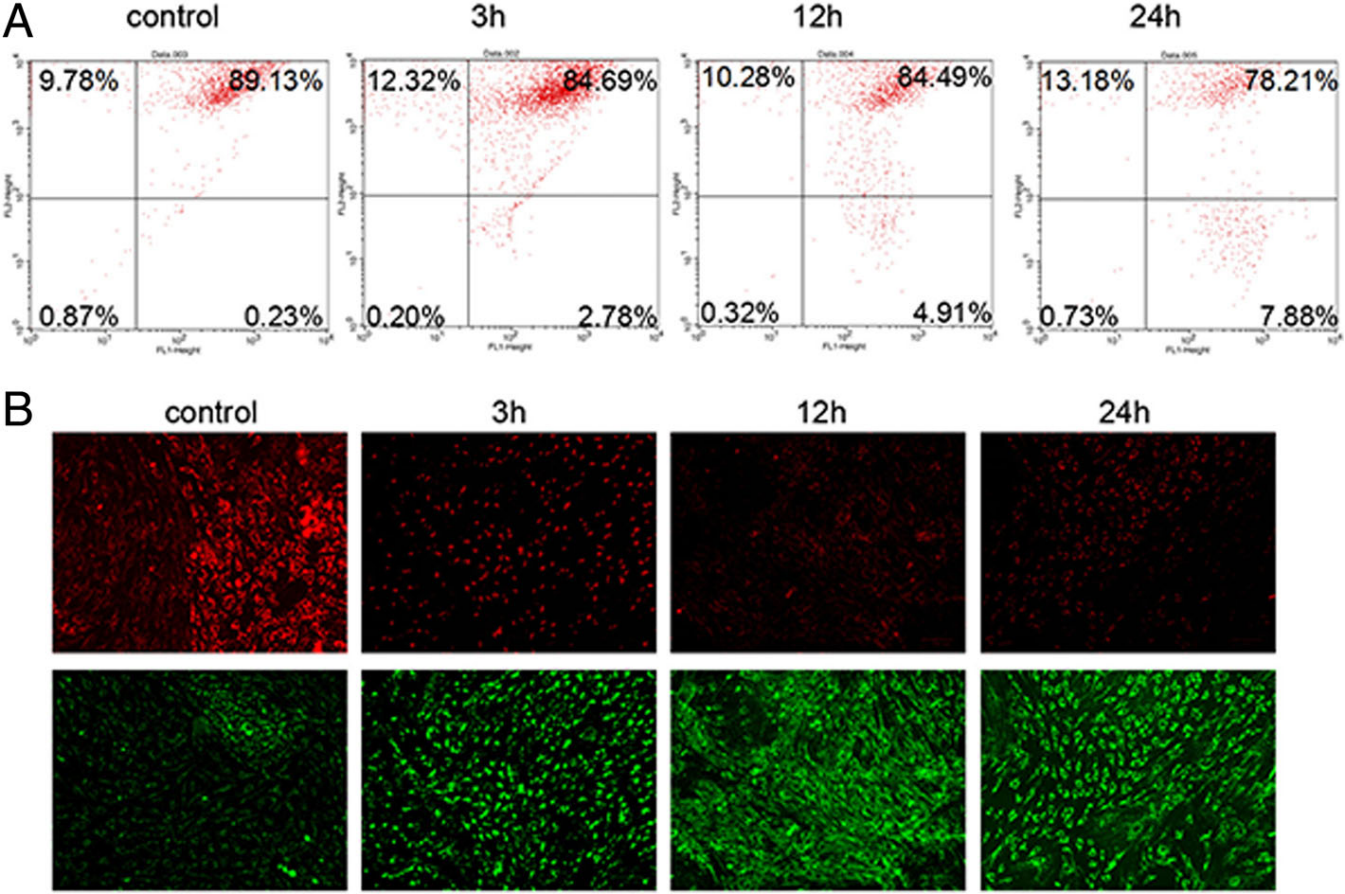
Mitochondrial membrane potential of rRECs in normal glucose medium after exposure to high glucose (20 mM). **a** Flow cytometry. **b** fluorescence microscopy

JC-1 dye was used as probe to measure mitochondrial membrane potential. The dye emitted green fluorescence as shown in the lower pane of Fig. 4b at lower membrane potential and did not accumulate in the mitochondrial matrix, while at higher membrane potential, it formed aggregates in the matrix and emitted red fluorescence (as shown in the upper panel of Fig. 4b). The measurements showed that when cultured in normal glucose medium, the percentage of the aggregate was 89.13%. The percentage decreased to 84.69% after the cells were exposed to 20 mM glucose for 3 h (Fig. 4a). At that time, green fluorescence was also observed (Fig. 4b). When the cells were exposed to 20 mM glucose for 12 h, they had 84.49% aggregate content with emission of green fluorescence, which was significantly lower than that of the control (*P* < 0.05). After 24 h exposure to high glucose, the percentage was even lower (78.21%, *P* < 0.05) after shifted to normal medium for 2 days as compared with control (Fig. 4a). These data suggest that high glucose reduces mitochondrial membrane potential even after the cells are shifted to normal glucose medium.

### High glucose increased ROS production

**Fig. 5:**
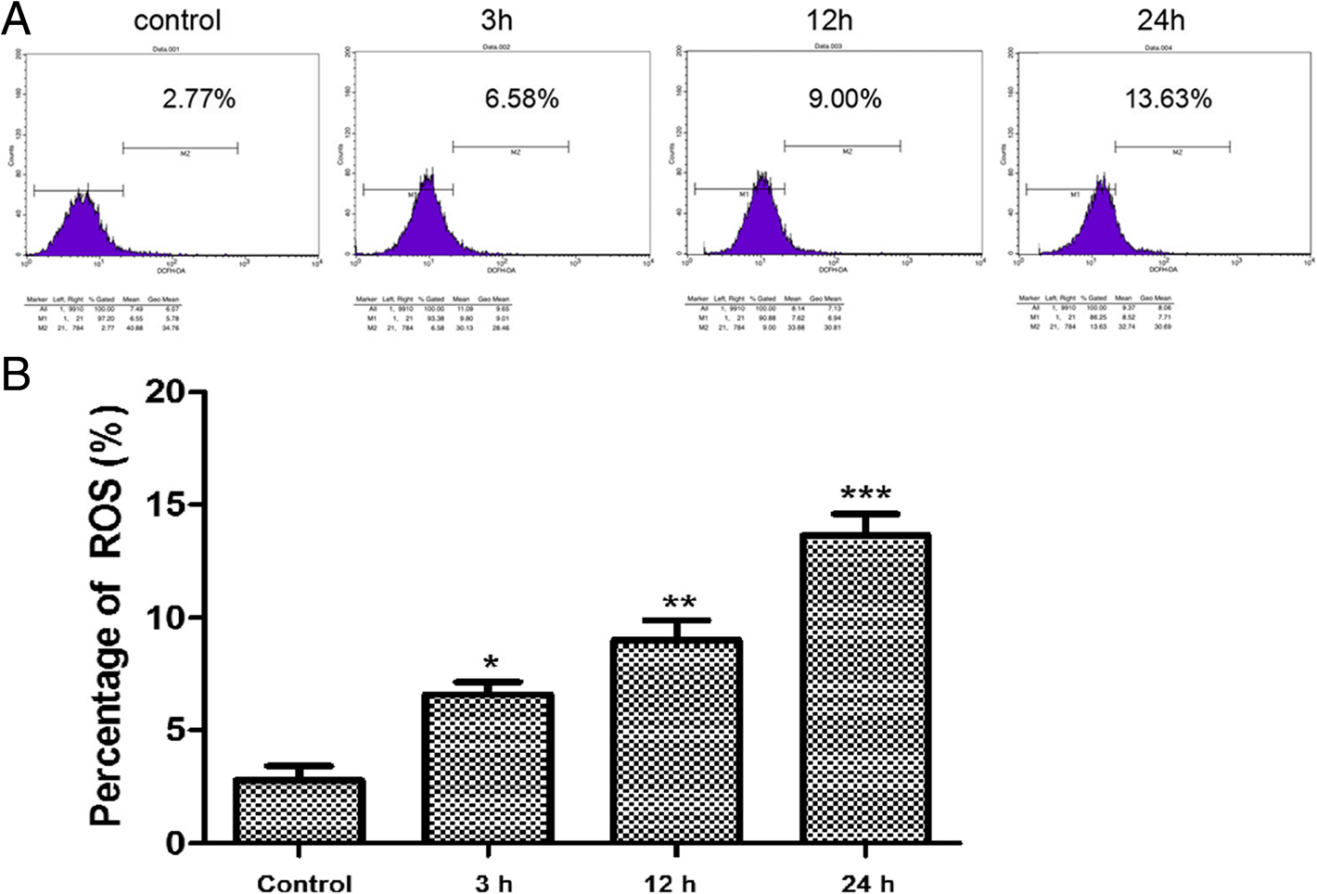
Levels of mitochondrial ROS detected by flow cytometry in rRECs in normal glucose medium after exposure to high glucose (20 mM). **a** flow cytometry; **b** percentage of ROS. * and ** denotes significant or highly significant difference vs control using one-way ANOVA with statistical significance set at a level of *P* < 0.05. Post-hoc multiple comparison between the groups was performed using S-N-K method. The bars represent std errors

DCFH-DA ROS assays showed that the ROS was 2.77% in the control cells and increased to 6.58% after 3 h exposure to high glucose medium and further to 9.00% after 12 h exposure to high glucose (*P* < 0.05 vs control and 3 h exposure). The percentage increased to 13.63% after 24 h exposure to high glucose (*P* < 0.05 vs control and 3 h exposure) (Fig. 5).

### High glucose increased apoptosis

**Fig. 6:**
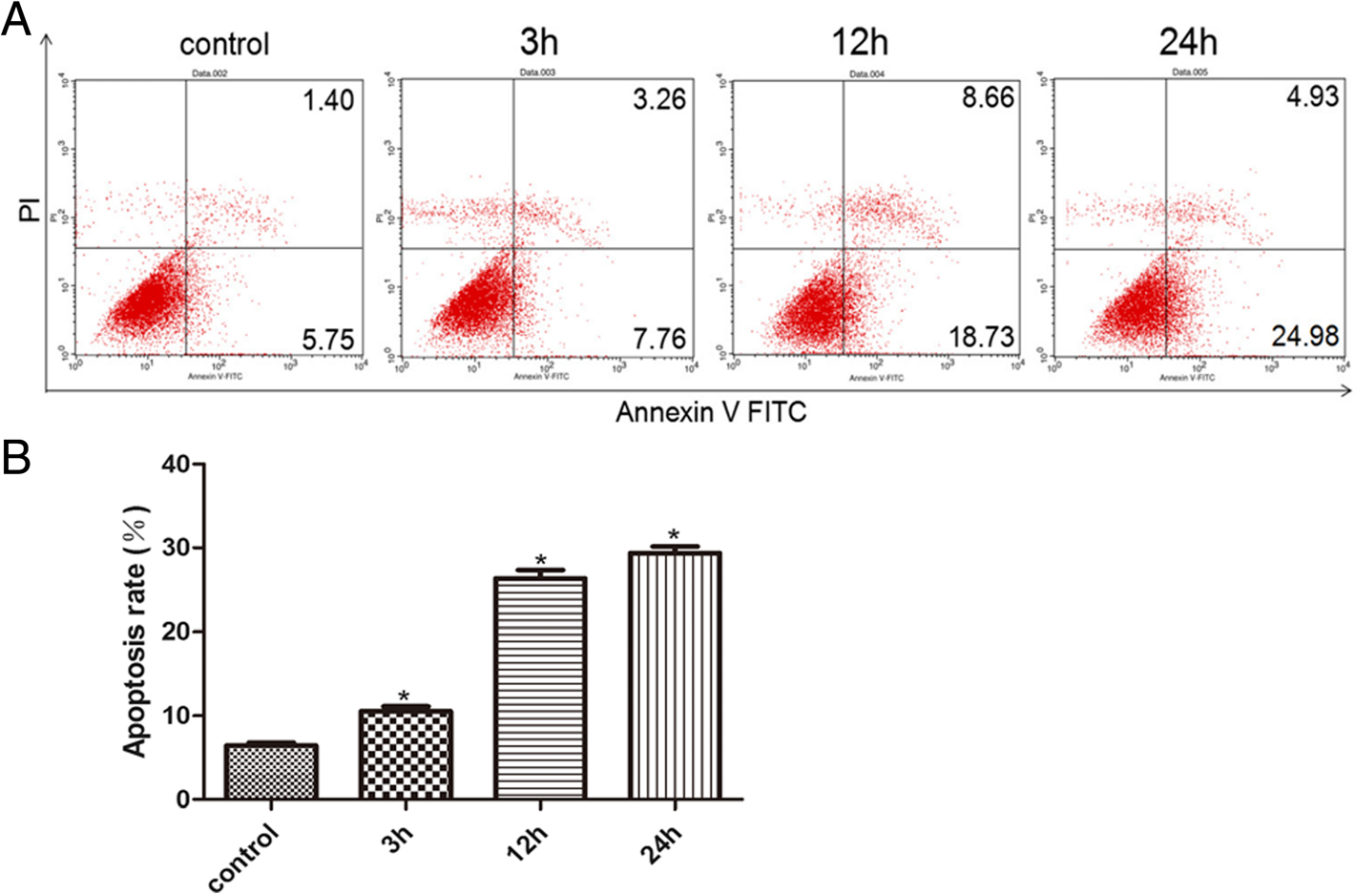
Apoptosis detected by flow cytometry in rRECs in normal glucose medium after exposure to high glucose (20 mM). **a** flow cytometry; **b** apoptotic rate. * and ** denotes significant or highly significant difference vs control. The bars represent std errors

Flow cytometry showed that the apoptosis rates increased significantly from 7.15% in the control group to 11.02, 27.39 and 29.91%, respectively, after 3, 12 and 24 h exposure to high glucose (*P* < 0.05) (Fig. 6). The increases were significantly different between the control and 3 h exposure (*P* < 0.05) or highly significantly different between the control and 24 h exposure (*P* < 0.01). Apoptosis rate after 24 h exposure was also significantly higher than after 3 h exposure (*P* < 0.05).

## Discussion

This study shows that high glucose induced mitochondrial damage as revealed by reduced membrane potential, increased apoptosis and ROS production even after the cells was shifted to normal glucose condition, suggesting that there is metabolic memory in the retinal cells.

Due to low patient compliance for long-term control of blood glucose, unclear diabetic DR pathogenesis, the lack of effective medicine for early intervention and poor outcomes of laser surgery and surgical operation for later stage patients [17], it is very important to have a better understand of early mechanism involved in DR pathogenesis to develop effective strategy for the prevention and treatment of the disease [18].

Clinically, DCCT (the Diabetes Control and Complications Trials) and EDIC (Epidemiology of Diabetes Interventions and Complications) have shown that there is metabolic memory in type 2 diabetes mellitus [19, 20], which is further confirmed in UKPDS (United Kingdom Prospective Diabetes Study) [21, 22]. In diabetic rat models, retinal mitochondrial dysfunction and oxidative stress still exist even after the blood glucose level has become normal [23, 24]. In addition, metabolic memory has also been found in isolated primary retinal cells [25].

Recent studies show that mitochondrial oxidative damage and dysfunction are associated with complications of nervous system diseases, diabetic cardiomyopathy and diabetes [26]. Mitochondrial oxidative damage and dysfunction in the heart of diabetic rats reduce the activity of mitochondrial respiratory chain-related enzymes [27]. Lee et al. found that high glucose inhibited the activity of mitochondrial electron transport chain complex in retinal cells and effectively promote the production of ROS and suppress the activation of NF-κB and TGF-β signaling pathways, suggesting that mitochondrial damage may result in ROS production in DR and ROS is responsible for the pathogenesis of DR [28]. Nishikawa et al. found that culturing vascular endothelial cells in high glucose medium led to ROS production and cell damage [29]. In this study we found that exposure to high glucose for 3 to 24 h resulted in time-dependent increase of ROS production as well as down-regulation of NAPDH-1 and CO-1, suggesting the high glucose could increase mitochondrial production of ROS and damage mitochondrial respiratory function. Since increase in ROS production was observed after the cells had been transferred to normal glucose condition, it is likely that the increased ROS production is due to metabolic memory.

DR is a microvascular complication of diabetes mellitus and is a microcirculation disorder. Early changes in DR include apoptosis of peripheral blood cells, microvascular occlusion, vascular leakage and microaneurysm [30]. REC is the first barrier to sense the changes in blood glucose and the main target of attack from diabetes complications. The dysfunction of REC is the common basis of microvascular complications including DR. DR pathogenesis is recognized to be associated with enhanced polyol pathway, increased glycosylated end products, activated *protein kinase C* and increased influx of glucose via hexosamine pathway [31]. The four seemly-independent pathways have been shown to be associated with a common high glucose-induced pathogenesis process - over-production of toxic mitochondrial ROS [32]. Our study also show that high glucose induced the overproduction of ROS in cultured RECs, resulting in mitochondrial oxidative damage and apoptosis.

In an early study, it was found that high glucose increased the level of 8-hydroxy-2′-deoxyguanosine after 3 h exposure of cell to high glucose, reduced mitochondrial membrane potential and increased ROS production after 12 h exposure, and increased apoptosis after 12 h exposure [33], suggesting that there might be mtDNA oxidative damage in early stage of DR, which results in further oxidative stress. For the first time, RECs were used to investigate DR metabolic memory at DNA damage level and to define the time frame within which metabolic memory occurs. Taking together, our findings indicate that mitochondrial oxidative stress is likely an important target for improving mitochondrial function. In the further, it would be important to define the optimal timepoint to block the vicious cycle of ROS production using RNAi technology to protect mitochondria from metabolic memory as a potential therapeutic option.

## Conclusion

Our data demonstrate that mitochondrial oxidative damage is likely to trigger metabolic memory via ROS overproduction that leads to diabetic retinopathy, and may be reduced using RNAi technology to attenuate the disease.

## References

[1] Weng JP, Bi Y (2015). Epidemiological status of chronic diabetic complications in China. Chin Med J.

[2] Galetovic D, Olujic I, Znaor L, Bucan K, Karlica D, Lesin M, Susac T (2013). The role of diabetic retinopathy in blindness and poor sight in Split-Dalmatia County 2000-2010. Acta Clin Croat.

[3] Vujosevic S, Midena E (2016). Diabetic retinopathy in Italy: epidemiology data and telemedicine screening programs. J Diabetes Res.

[4] Tracey ML, McHugh SM, Fitzgerald AP, Buckley CM, Canavan RJ, Kearney PM (2016). Trends in blindness due to diabetic retinopathy among adults aged 18-69years over a decade in Ireland. Diabetes Res Clin Pract.

[5] Alcubierre N, Rubinat E, Traveset A, Martinez-Alonso M, Hernandez M, Jurjo C, Mauricio D (2014). A prospective cross-sectional study on quality of life and treatment satisfaction in type 2 diabetic patients with retinopathy without other major late diabetic complications. Health Qual Life Outcomes.

[6] Zhang L, Chen B, Tang L (2012). Metabolic memory: mechanisms and implications for diabetic retinopathy. Diabetes Res Clin Pract.

[7] Lee C, An D, Park J (2016). Hyperglycemic memory in metabolism and cancer. Horm Mol Biol Clin Investig.

[8] Bhatt MP, Lee YJ, Jung SH, Kim YH, Hwang JY, Han ET, Park WS, Hong SH, Kim YM, Ha KS (2016). C-peptide protects against hyperglycemic memory and vascular endothelial cell apoptosis. J Endocrinol.

[9] Kowluru RA (2013). Mitochondria damage in the pathogenesis of diabetic retinopathy and in the metabolic memory associated with its continued progression. Curr Med Chem.

[10] Kukat A, Dogan SA, Edgar D, Mourier A, Jacoby C, Maiti P, Mauer J, Becker C, Senft K, Wibom R (2014). Loss of UCP2 attenuates mitochondrial dysfunction without altering ROS production and uncoupling activity. PLoS Genet.

[11] Wu H, Jiang C, Gan D, Liao Y, Ren H, Sun Z, Zhang M, Xu G (2011). Different effects of low- and high-dose insulin on ROS production and VEGF expression in bovine retinal microvascular endothelial cells in the presence of high glucose. Graefes Arch Clin Exp Ophthalmol.

[12] Li SY, Fu ZJ, Lo AC (2012). Hypoxia-induced oxidative stress in ischemic retinopathy. Oxidative Med Cell Longev.

[13] Giacco F, Brownlee M (2010). Oxidative stress and diabetic complications. Circ Res.

[14] Li N, Karaca M, Maechler P (2017). Upregulation of UCP2 in beta-cells confers partial protection against both oxidative stress and glucotoxicity. Redox Biol.

[15] Morigi M, Angioletti S, Imberti B, Donadelli R, Micheletti G, Figliuzzi M, Remuzzi A, Zoja C, Remuzzi G (1998). Leukocyte-endothelial interaction is augmented by high glucose concentrations and hyperglycemia in a NF-kB-dependent fashion. J Clin Invest.

[16] Livak KJ, Schmittgen TD (2001). Analysis of relative gene expression data using real-time quantitative PCR and the 2(−Delta Delta C(T)) method. Methods.

[17] Santos JM, Kowluru RA (2011). Role of mitochondria biogenesis in the metabolic memory associated with the continued progression of diabetic retinopathy and its regulation by lipoic acid. Invest Ophthalmol Vis Sci.

[18] Engerman RL, Kern TS (1987). Progression of incipient diabetic retinopathy during good glycemic control. Diabetes.

[19] Bianchi C, Del Prato S (2011). Metabolic memory and individual treatment aims in type 2 diabetes--outcome-lessons learned from large clinical trials. Rev Diabet Stud.

[20] De Felice FG, Ferreira ST (2014). Inflammation, defective insulin signaling, and mitochondrial dysfunction as common molecular denominators connecting type 2 diabetes to Alzheimer disease. Diabetes.

[21] Pirola L, Balcerczyk A, Okabe J, El-Osta A (2010). Epigenetic phenomena linked to diabetic complications. Nat Rev Endocrinol.

[22] Ranjit Unnikrishnan I, Anjana RM, Mohan V (2011). Importance of controlling diabetes early--the concept of metabolic memory, legacy effect and the case for early insulinisation. J Assoc Physicians India.

[23] Kowluru RA, Chan PS (2010). Metabolic memory in diabetes - from in vitro oddity to in vivo problem: role of apoptosis. Brain Res Bull.

[24] Zhong Q, Kowluru RA (2011). Epigenetic changes in mitochondrial superoxide dismutase in the retina and the development of diabetic retinopathy. Diabetes.

[25] Zhao S, Li J, Wang N, Zheng B, Li T, Gu Q, Xu X, Zheng Z (2015). Feno fi brate suppresses cellular metabolic memory of high glucose in diabetic retinopathy via a sirtuin 1-dependent signalling pathway. Mol Med Rep.

[26] Sharma K, Karl B, Mathew AV, Gangoiti JA, Wassel CL, Saito R, Pu M, Sharma S, You YH, Wang L (2013). Metabolomics reveals signature of mitochondrial dysfunction in diabetic kidney disease. J Am Soc Nephrol.

[27] Casalena G, Daehn I, Bottinger E (2012). Transforming growth factor-beta, bioenergetics, and mitochondria in renal disease. Semin Nephrol.

[28] Moreira PI, Rolo AP, Sena C, Seica R, Oliveira CR, Santos MS (2006). Insulin attenuates diabetes-related mitochondrial alterations: a comparative study. Med Chem.

[29] Chacko BK, Reily C, Srivastava A, Johnson MS, Ye Y, Ulasova E, Agarwal A, Zinn KR, Murphy MP, Kalyanaraman B (2010). Prevention of diabetic nephropathy in Ins2(+/)(−)(AkitaJ) mice by the mitochondria-targeted therapy MitoQ. Biochem J.

[30] Fraser-Bell S, Symes R, Vaze A (2017). Hypertensive eye disease: a review. Clin Exp Ophthalmol.

[31] Otto-Buczkowska E, Machnica L (2010). Metabolic memory - the implications for diabetic complications. Endokrynol Pol.

[32] Nishikawa T, Edelstein D, Du XL, Yamagishi S, Matsumura T, Kaneda Y, Yorek MA, Beebe D, Oates PJ, Hammes HP (2000). Normalizing mitochondrial superoxide production blocks three pathways of hyperglycaemic damage. Nature.

[33] Xie L, Zhu X, Hu Y, Li T, Gao Y, Shi Y, Tang S (2008). Mitochondrial DNA oxidative damage triggering mitochondrial dysfunction and apoptosis in high glucose-induced HRECs. Invest Ophthalmol Vis Sci.

